# A MEMS grating modulator with a tunable sinusoidal grating for large-scale extendable apertures

**DOI:** 10.1038/s41378-025-00894-7

**Published:** 2025-03-03

**Authors:** Datai Hui, Dongpeng Li, Binbin Wang, Yongqian Li, Jiaqian Ding, Laixian Zhang, Dayong Qiao

**Affiliations:** 1 Key Laboratory of Micro/Nano Systems for Aerospace, Ministry of Education, Northwestern Polytechnical University, 710072 Xi’an, China; 2 Research & Development Institute of Northwestern Polytechnical University, 315103 Ningbo, China; 3 Key Laboratory of Intelligent Space TTC&O, Ministry of Education, Space Engineering University, 101416 Beijing, China

**Keywords:** Micro-optics, Electrical and electronic engineering

## Abstract

Microelectromechanical system (MEMS) grating modulators enable versatile beam steering functions through the electrostatic actuation of movable ribbons. These modulators operate at ultrahigh frequencies in the hundred kHz range, and their micromirror-free configuration simplifies the fabrication process and reduces costs compared to micromirror-based modulators. However, these modulators are limited in their optical efficiency and aperture. Here, we present a MEMS grating modulator with a notably extendable aperture and a high optical efficiency that benefits from the adoption of a tunable sinusoidal grating. Instead of end-constrained movable ribbons, we constrain the MEMS grating modulator through broadside-constrained continuous ribbons. The end-free grating enables improved scalability along the ribbons, and the continuous sinusoidal surface of the grating allows an increased fill factor. As an example, we experimentally demonstrate a MEMS grating modulator with a large-scale aperture of 30 × 30 mm and an optical efficiency of up to 90%. The modulation depth enables intensity modulation across a broad wavelength range from 635 to 1700 nm. The experimental results demonstrate that the reported modulator has a mechanical settling time of 1.1 μs and an extinction ratio of over 20 dB. Furthermore, it offers a dynamic modulation contrast of over 95% within a 250 kHz operating frequency and achieves full modulation within a field of view (FOV) of ±30°. The reported MEMS grating modulator holds promise for application in high-speed light attenuation and modulating retroreflector free-space optical (MRR-FSO) communication systems. Our device also paves new ways for future high-speed, energy-efficient, and cost-effective communication networks.

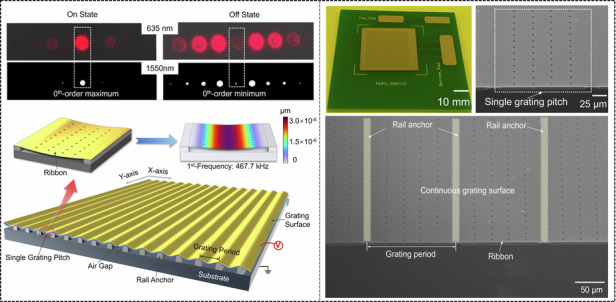

## Introduction

Over the past few decades, the continuous development of optical techniques has significantly promoted their more efficient and effective applications in various fields, such as in modern life scenarios^1,2^, optical communication science^3^, and remote sensing^4,5^. Various innovative optical modulation technologies have been proposed and investigated to meet the increasing demand for different applications. These technologies include acousto-optic modulators (AOMs)^6^, liquid crystal (LC) modulators^7^, electro-optic modulators (EOMs)^8^, multiple quantum well (MQW) modulators^9^, and microelectromechanical system (MEMS) modulators^10^. Among these, MEMS optical modulators integrate multiple electrical and mechanical functionalities, allowing a wider wavelength range, a higher modulation contrast^11^, and lower susceptibility to performance degradation due to temperature variations^12^. Additionally, MEMS optical devices can withstand high optical power and enable polarization-independent operation. Due to these characteristics, MEMS modulators have attracted wide research and industrial interest for various applications in free-space optical (FSO) communication^13,14^, light detection and ranging^15,16^, adaptive optics systems^17,18^, and other remote sensing applications^19^.

Therefore, various innovative MEMS optical modulators have been well developed and play essential roles in numerous functional optical systems, including digital micromirror devices (DMDs)^20–22^, deformable micromirror (DM) arrays^17^, and MEMS diffraction gratings^23^. The micromirror-type devices integrate multiple electrical and mechanical functionalities, in which the intensity and/or phase of light are manipulated through microsized movable structures^24^. The deflection-driven micromirror arrays reflect light at twice the mechanical deflection angle and offer a variety of reconfigurable mirror surfaces for spatial light modulation^25,26^ and wavefront correction in microscale pixels^27^. The micromirror-based MEMS optical modulators commonly feature multiple-layer configurations with actuators positioned beneath the micromirrors. The large moment of inertia of the movable micromirror determines that the operating frequency is within 50 kHz^21,26^.

As a different MEMS optical modulator without a micromirror, MEMS gratings have the benefit of a higher operating frequency because of the operational speed of optical signals^23^. MEMS gratings redistribute light energy among different diffraction orders by employing repeating elementary structures^28,29^, which steer a wavefront of interest to form spectral lines or direct it to a specified angle. Additionally, their two-layer configuration simplifies the fabrication process. Therefore, many efficient MEMS grating architectures have been explored for combining with advanced MEMSs to meet the demands for the functions and properties of portable optical systems. For example, the optimization of high-contrast grating (HCG) structures^29^ and the configuration of 1D-rectangular grating arrays of movable and static ribbons^30^ have increased their operational frequency to the MHz level. However, owing to the bending deformation of the movable ribbons, the configured grating profile cannot achieve perfect diffraction, resulting in an overall optical efficiency of ~70%^29,30^. To mitigate bending deformation and promote optical efficiency, various movable structural optimizations have been investigated. Integrating flexible hinges with movable structures represents one of the most effective strategies^31–33^. The flexible hinge reduces the bending deformation between the movable ribbons and their support so that they can perform a perfect piston motion under actuation, thus enlarging the diffraction area^34,35^. However, the main drawback of an integrated flexible hinge is that it deteriorates the dynamic performance of MEMS grating modulators. The resonant frequency of the resulting optical system is determined by the mechanical coupling among the movable ribbons and the differential stiffness of the bending deformation of the suspended hinges. The stiffness of the flexible hinge structure deteriorates the dynamic performance of MEMS grating modulators, thus limiting the operating frequency to dozens of kHz^31–33,36^ while decreasing the overall optical efficiency to less than 70% because of the diminished fill rate of the reflective surface. Advanced manufacturing technologies have enabled these grating modulators to be used in a wide range of application scenarios for the miniaturization of optical systems. For some optical instruments, particularly those used in remote sensing and FSO communication^14,37^, large aperture sizes are required to obtain maximum beam areas, especially for devices operating at higher power levels. Producing the large apertures required for high-power devices is challenging since the surface shape over the entire area should be maintained within less than one optical wavelength. The electrostatic pull-in effect limits the usable displacement to half of the gaps between parallel-plate electrodes. Although increasing the length of movable ribbons can expand the aperture size, this also decreases the resonant frequency, consequently reducing the overall operating frequency of the modulator. To date, no modulator can simultaneously achieve all the desired characteristics, including a large aperture size, a high optical efficiency, wideband wavelengths, and a high resonant frequency (corresponding to a fast response time). To date, the aperture size of MEMS grating modulators is limited to ten square millimeters^29,33,36^.

Here, we report a MEMS grating modulator with a notably large aperture and a high optical efficiency that benefits from the adoption of a tunable sinusoidal grating. The tunable sinusoidal grating is configured using a grating pitch array. Each pitch is designed with a double-clamped beam to generate deflection motion under an electrostatic force. Unlike end-constrained movable ribbons, the movable ribbons of the proposed MEMS grating modulator are constrained along their broadside by rail anchors. This enables enhanced scalability of the modulator along the ribbons while maintaining its resonant frequency. As an example, we demonstrate a modulator with an aperture of over 30 × 30 mm. In addition, a continuous sinusoidal grating profile rather than a rectangular discrete grating is adopted to modulate the diffraction intensity, with an optical efficiency greater than 90%. Moreover, the configuration of the release holes on the grating surface is optimized to achieve a dynamic response frequency higher than 250 kHz. A comparison of key properties between the sinusoidal grating-based modulator reported here and other relevant works is summarized in Table S1 of Supplementary Information.

## Results

### Device configuration and principle

**Fig. 1:**
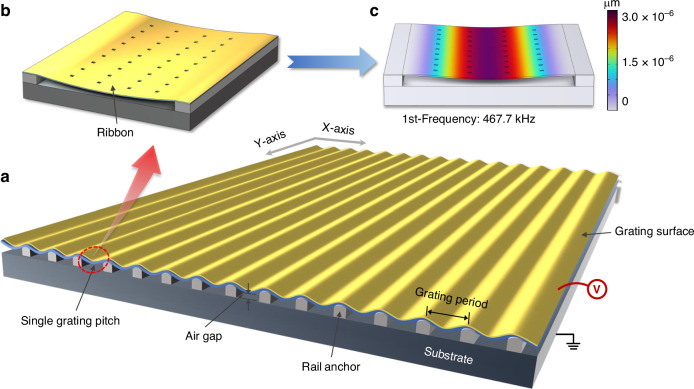
Schematic diagram of the proposed MEMS grating modulator. **a** Structure of the modulator. **b** Single grating pitch of the MEMS grating modulator. **c** First resonant mode and its frequency for a single grating pitch

The proposed MEMS grating modulator is constructed with a tunable grating pitch array, in which each pitch is designed with a broadside-constrained continuous ribbon to generate deflection deformation (as shown in Fig. 1). These grating pitches are simultaneously actuated to reconfigure a tunable sinusoidal grating with the desired modulation depth. This is achieved through electrostatic parallel-plate actuation, in which the substrate and the grating surface serve as the bottom and top electrodes, respectively. The grating surface is connected to the substrate through periodically arranged insulated rail anchors. The entire grating surface is designed as a continuous surface to maintain a good sinusoidal profile during actuation. This design also enables the modulator to achieve a surface fill factor of over 96.6%. In the tunable sinusoidal grating, the grating period refers to the distance between two consecutive peaks of the grating under actuation. In our current design, the exact value of the grating period is 150 µm. Exploiting the unidirectional equal stiffness of the grating pitch, the pitch length can be extended to create a large-scale aperture by arranging the pitches in a one-dimensional array, which enables the scalability of the grating modulator. The continuous sinusoidal grating with an extendable large aperture size of the proposed optical modulator allows a higher utilization ratio of light energy, particularly in long-distance FSO communication applications^35^. Importantly, the overall resonant frequency of the entire modulator is determined by a single grating pitch. Each grating pitch is constructed with a broadside-constrained continuous ribbon. The thickness and width of the ribbon are designed as 1.0 and 135 μm, respectively. These structural parameters and configuration of the release holes through its grating surface are adjusted to achieve the resonant frequency of interest under an electrostatic force. The air gap is determined by the thickness of the silicon oxide in the SOI wafer. The commercial SOI wafer with a silicon oxide of 3.0 μm was selected to ensure a broad working wavelength range from 530 to 1700 nm. This gap ensures that sufficient displacement exists at the longest wavelength without being affected by the pull-in effect. A strategically arranged set of through-holes on the grating surface (Fig. 1b) enhances the flowability of air under the continuous grating pitch. These through-holes also modulate the necessary damping action to minimize unexpected oscillations and reduce the dynamic response time. The resonant frequencies and modes of the optical modulator were analyzed via the finite element method (FEM). Figure 1c shows the first resonant mode of a single grating pitch. The desired first-order mode shows flexural deformation at a resonant frequency of 467.7 kHz. Further optimization of the resonant frequency of the grating structure indicates its ability to operate at a microsecond response time.

**Fig. 2:**
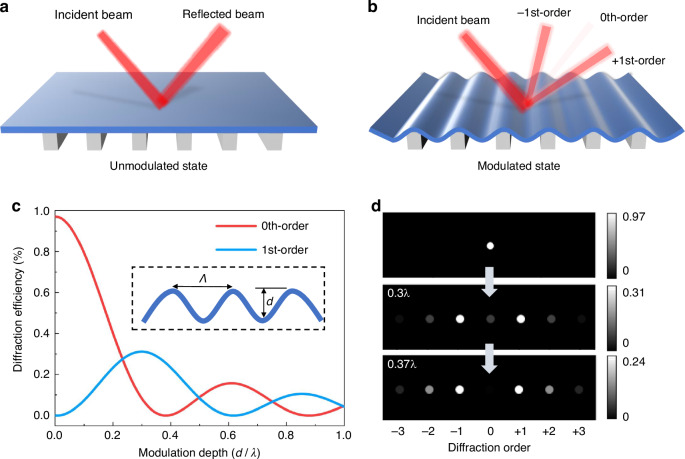
Modulation principle and diffraction behavior of the MEMS grating modulator with a tunable sinusoidal grating. Surface profiles of the unmodulated (**a**) and modulated (**b**) modulator. The reflected beam is modulated into diffraction orders because of the deformable bending of the continuous sinusoidal gratings. **c** Diffraction efficiencies of a sinusoidal grating and its diffraction pattern. Zeroth- and first-order diffraction efficiencies versus modulation depth. **d** Simulated diffraction patterns with varying modulation depth

The optical modulator operates based on the grating diffraction theory. As illustrated in Fig. 2a, in the unmodulated state, the grating surface initially approximates an optically flat surface to reflect most of the incident light energy. In the modulated state, the movable part of the grating bends downwards towards the substrate, in which the grating exhibits a sinusoidal profile, and a substantial amount of light is redirected to higher orders (Fig. 2b). The diffraction angle for each order depends on both the grating period and the operating wavelength. At normal incidence, the diffraction angle of each diffraction order is expressed as:

1 $${\theta }_{m}={{\rm{s}}{\rm{in}}}^{-1}\left(\frac{m\lambda }{\Lambda }\right)$$

where $m$ is the diffraction order, $\lambda$ is the light wavelength, and $\varLambda$ is the grating period. Since the grating period is 150 µm in the current design, the diffraction angle of each order is determined solely by the operating wavelength and increases monotonically with wavelength. At the specific wavelengths of 635, 1050, and 1550 nm, the first diffraction angles of our grating are ±0.24°, ±0.40°, and ±0.56, respectively.

For the wavelength of interest, the desired diffraction efficiency for a specific order depends on the modulation depth of the sinusoidal grating. The modulation depth, relative to the wavelength $\lambda$, corresponds to an effective displacement *d* of the sinusoidal grating due to deformable bending. Typically, the zeroth- and first-order beams are emphasized because of their relatively high light energy efficiency. Therefore, to verify the feasibility of the sinusoidal grating-based optical modulator, we first conducted theoretical calculations and numerical simulations to determine the dependence of the diffraction efficiency on the modulation depth. The diffraction efficiency of a sinusoidal profile grating can be described by the Bessel function^38^:

2 $${\eta }_{m}={J}_{m}^{2}\left\{\frac{\pi d}{\lambda }\left(\cos \alpha +\cos {\alpha }_{m}\right)\right\}$$

where ${\eta }_{m}$ is the diffraction efficiency of the *m*th diffraction order and ${J}_{m}$ is the *m*th-order Bessel function of the first kind. *d* represents the modulation depth of the grating, *λ* represents the wavelength of the incident light, $\alpha$ denotes the incident angle, and ${\alpha }_{m}$ indicates the angle of the *m*th diffraction order.

The calculated diffraction efficiencies of the zeroth- and first-order diffraction components are shown in Fig. 2c. Figure 2d shows the simulation results of the diffraction pattern with varying modulation depth. The maximum diffraction efficiency of the first-order reflected beam approaches 31% for a modulation depth of 0.3$\lambda$ (blue curve in Fig. 2c). The maximum diffraction efficiency of the zeroth-order reflection is ~97%, and no high-order diffraction is observed for a flat surface (red curve in Fig. 2c). When the modulation depth increases to 0.37 $\lambda$, the diffraction efficiency of the zeroth-order beam decreases to almost zero. In the visible to near-infrared wavelength range, the zeroth-order beam can provide the highest modulation contrast for intensity modulation. By adjusting the modulation depth from 0 to 0.37 *λ*, a continuous variation in the zeroth-order efficiency from 97% to near zero can be achieved.

### Device fabrication

**Fig. 3:**
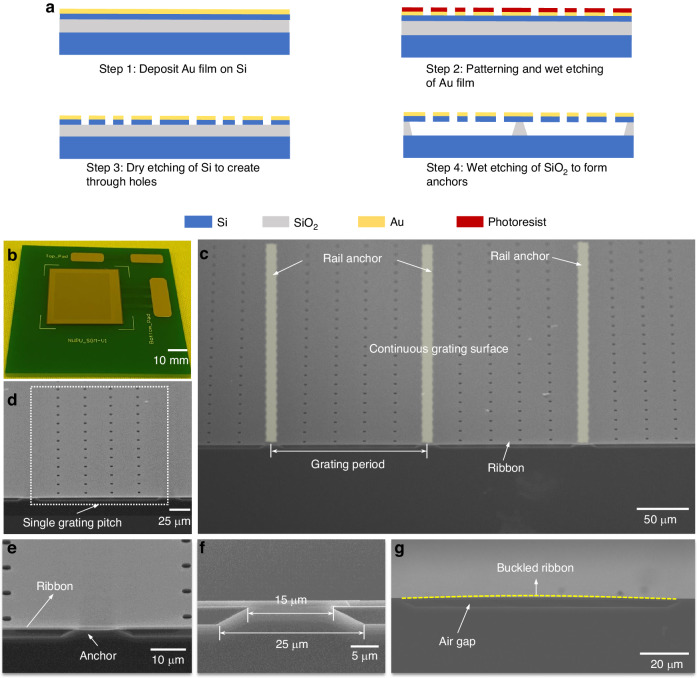
Fabrication processes and fabricated MEMS grating modulator. **a** Fabrication processes of the MEMS grating modulator. **b** Photograph of a fabricated modulator bonded to a PCB. **c** SEM image of the continuous grating surface. **d** SEM image of a single grating pitch. **e** SEM image of the cross-section of a grating pitch. **f** Detailed structural hierarchy of an insulated anchor. **g** Cross-sectional view of the air gap between the grating surface and the substrate insulated by anchors

The MEMS grating modulator was fabricated via a two-mask micromachining process with a double-polished silicon-on-insulator (SOI) wafer. The fabrication processes are shown in Fig. 3a. First, a gold film with a thickness of 50 nm was physically vapor deposited on the SOI device layer to enhance the reflectivity of the modulator surface (step 1). The gold film was lithographically patterned and then wet-etched to expose a portion of the surface of the SOI device layer for subsequent dry etching (step 2). Dry etching was performed via an inductively coupled plasma (ICP) etching process to form a pattern of through-hole arrays through the SOI device layer (step 3). Finally, the silicon dioxide layer was partially etched using hydrofluoric acid by a wet release process s (step 4). The residual silicon dioxide acts as a rail anchor, and the grating period is defined by the period of the through holes.

Ensuring reproducibility relies on the control of the wet etching process parameters, including the concentration of hydrofluoric acid, release time, and temperature variation. These parameters determine the ratio between the dimensions of the rail anchor and the grating ribbon, which in turn affects the profile shape of the tunable sinusoidal grating and its mechanical properties. The challenge in maintaining uniformity across larger devices is release stiction due to the wet etching. After silicon dioxide removal, the restoring force of the ribbons cannot overcome capillary forces caused by residual etching liquid with high surface energy^39,40^. To address this, we conducted a deionized (DI) water combined with isopropyl alcohol (IPA) rinse procedures^41^ to prevent stiction. Specifically, after release from hydrofluoric (HF) acid, the device was first immersed in DI water to replace the residual etching liquid. The DI water was replaced in 5 min and at least three times to complete the removal of the etching liquid. Next, the device was immersed in IPA solution for the second step, where IPA with even lower surface energy to replace the DI water. Finally, the device was transferred to a preheated oven for rapid drying, ensuring quick evaporation of any remaining IPA droplets. Using this method, no release stiction was observed on the devices across the entire wafer in our all experiments.

**Fig. 4:**
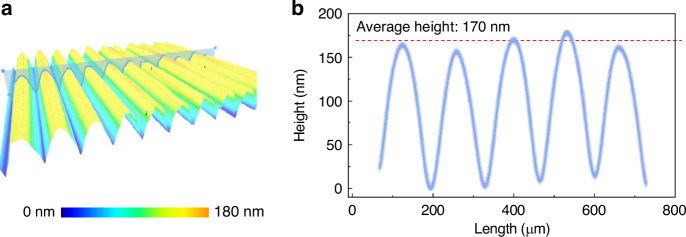
Grating topography measured by a white light interferometer. **a** 3D morphology of the grating surface. **b** Upward buckling deformation of the grating surface

Figure 3b displays a photograph of a fabricated modulator chip bonded to a printed circuit board (PCB). In this example, the fabricated MEMS grating modulator chip includes hundreds of grating pitches with a size of 150 μm, and the optical aperture is extended to 30 mm × 30 mm (900 mm^2^). The high surface fill factor of 96.6% and the gold film coated on the grating surface enable the modulator to achieve an optical efficiency of over 90%. Scanning electron microscopy (SEM) images of the fabricated MEMS grating modulator are shown in Fig. 3c–g. Figure 3c shows a tilted SEM image of the continuous grating surface and a single grating pitch. Figure 3d, e show zoomed-in images of a grating pitch and an insulated anchor, and their cross-sectional images are shown in Fig. 3f, and it can be observed that the shape of the insulated anchor appears to be trapezoidal. The top and bottom widths of each insulated anchor are ~15 and 25 μm, respectively. The width difference results from the different etching rates of the hydrofluoric acid solution along the lower interface and the upper interface between the single-crystal silicon and silicon dioxide^42^. However, the smaller top width than the bottom width of the anchors is expected to facilitate good formation of a sinusoidal profile, which greatly benefits the optical efficiency and modulation contrast. It is obvious, noting from Fig. 3g, that the ribbon exhibits an initial upward buckling deformation, which could be induced by an intrinsic compressive stress in the SOI device layer. To further investigate the details of the morphology, the upward deformation was measured by a white light interferometer. As shown in Fig. 4a, the 3D morphology indicates that the surface of each grating ribbon displays an upward buckling deformation. The average initial upward deformation is ~170 nm, as shown in Fig. 4b.

### Device characterization

**Fig. 5:**
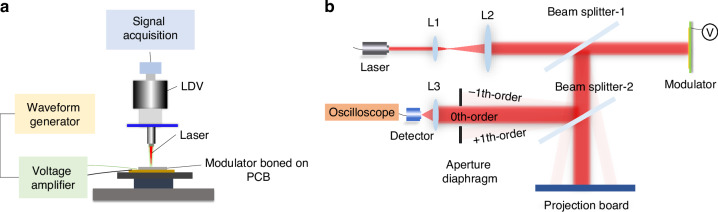
Measurement of the fabricated modulator. **a** LDV-based system for characterizing the electromechanical properties. **b** Intensity characterization system for examining the optical performance

Figure 5 presents the method used to characterize the fabricated modulator. Figure 5a shows the electromechanical performance measurement setup using a laser doppler vibrometer (LDV). A narrow linewidth laser was focused on the middle of the grating pitch to capture the maximum displacement. The modulator was bonded to a PCB and actuated by a voltage amplifier. As illustrated in Fig. 5b, an optical setup was constructed to characterize the optical performance of the MEMS grating modulator. The intensity-based characterization system consisted of a low-power narrow laser for illumination. Two collimating lenses with different focal lengths were placed on the conjugate focal plane to expand the point light into a plane wave. The first 50/50 beam splitter was mounted at a 45-degree angle to transmit the input plane wave and reflect the modulated beam from the modulator. The other beam splitter was placed parallel to the first beam splitter to project the far-field pattern onto a board and reflect the modulated beam into the detector. An aperture diaphragm was set in front of the detector to block all high-order beams except for the zeroth-order beam. By placing a PIN photodiode detector or a power meter at the Fourier plane of the lens, the changes in the light intensity at the 0th diffraction order could be monitored as the modulator transitioned between the modulated and unmodulated states. Both the static and dynamic properties of an optical modulator were evaluated in this optical setup.

**Fig. 6:**
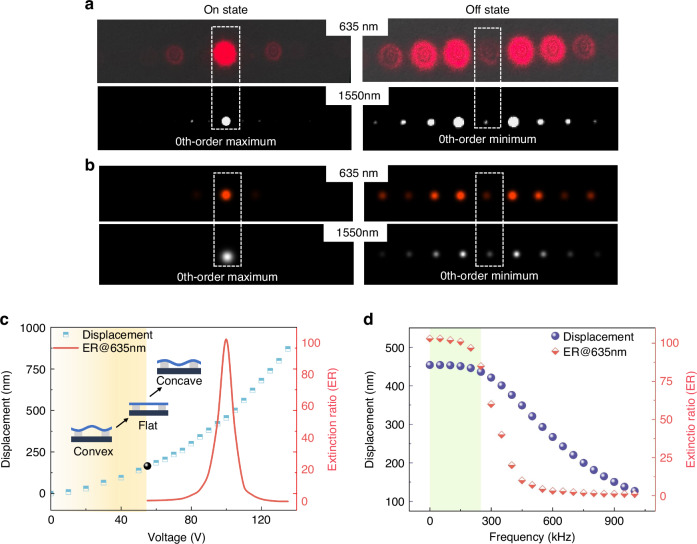
Statics performance of the MEMS grating modulator. Far-field diffraction patterns in the **a** experimental results for the “on” and “off” states at wavelengths of 635 and 1550 nm show high consistency with **b** simulation results. The significant difference in the 0th-order spot between the two states highlights the superior modulation contrast of the MEMS grating modulator. **c** Displacement and corresponding extinction ratio (ER@635 nm) of the modulator under varying voltages. As the voltage increases, the grating profiles change from convex (unmodulated) to flat (“on” state) and finally to concave (“off” state). **d** The frequency response indicates a critical damping or overdamped response, where both displacement and the extinction ratio decrease with increasing frequency but remain stable below 250 kHz, enabling the modulator to operate effectively and achieve nearly full modulation within this frequency range

To first provide an intuitive optical characterization, laser sources with wavelengths of 635 and 1550 nm were used to analyze the far-field diffraction pattern and estimate the “on” and “off” state actuation. Figure 6a-left displays the diffracted beam spots in the “on” state with an actuation voltage of about 55 V, and the maximum intensity is shown by the 0th-order beam spot. With an increase in the applied voltage to 100 and 125 V, the “off” state is achieved for the 635 and 1550 nm wavelengths, respectively, as shown in Fig. 6a-right. In the “off” state, the intensity of the zeroth-order beam decreases, whereas the intensity of higher diffraction orders increases in the off-axis direction. The significant difference in the 0th-order intensity between the “on” and “off” states reflects the extinction ratio and modulation contrast of our MEMS optical modulator. Figure 6b displays the “on/off” state diffraction patterns simulated at wavelengths of 635 and 1550 nm. The simulation patterns are highly consistent with the experimental results. The patterns obtained in the experiment have many more high-order spots than the simulation patterns. The extra high-order spots are attributed to the through-hole arrays through the grating surface. These additional phase gratings result in these high-order diffraction spots. These high-order beams can be eliminated by further optimizing the gap between the modulator surface and substrate to achieve a π phase difference.

The static response of our fabricated modulator, in which both the extinction ratio and the displacement of the grating pitch were measured, is shown in Fig. 6c. The extinction ratio is a key metric to characterize the performance of optical modulators. The extinction ratio (ER) is defined as the ratio of the optical power reflected from the surface of the modulator when it works in the “on” state to the reflected optical power corresponding to the “off” state. The definition can be expressed by the following formula:

3 $${{\mathrm{Extinction}}\; {\mathrm{Ratio}}}=\frac{{P}_{{\rm{On}}}}{{P}_{{\rm{Off}}}}$$

where ${P}_{{\rm{On}}}$ is the optical power corresponding to the “on” state, and *P*_Off_ corresponding to the “off” state. The extinction ratio quantifies the contrast between two states. A higher extinction ratio allows the modulator to clearly distinguish between two states. Here, the intensity in the zeroth-order beam was selected to calculate the extinction ratio because it had the highest throughput. The extinction ratio is defined as the maximum-to-minimum power ratio of the zeroth-order beam. The optical power through an aperture diaphragm was measured by a standard photodiode at a wavelength of 635 nm. As illustrated in Fig. 6c, the grating surface profiles can be categorized into three states with increasing voltage. In the unenergized state, the grating surface exhibits convex deformation induced by the intrinsic pressure stress. When a voltage is applied, the electrostatic attraction forces the surface profile to transition from a convex to the flat surface. A flat surface profile is achieved at 55 V, where the light intensity of the zeroth-order reflection reaches its maximum, which is defined as the “on state”. As the voltage continues to increase, the grating surface undergoes concave deformation with an increase in the modulation depth, leading to a significant increase in the extinction ratio. At a voltage of 100 V, the optimal modulation depth for the zeroth-order beam at 635 nm is achieved, resulting in the zeroth-order beam reaching its weakest intensity, which is defined as the “off state”. The corresponding extinction ratio exceeds 100 (>20 dB) at this point. As the optimal modulation depth is exceeded with a further increase in the voltage, the increased intensity of the 0^th^-order beam leads to a notable decrease in the extinction ratio. Figure 6d also shows that the displacement can cover wavelengths of up to 1700 nm within 140 V, corresponding to a modulation depth of 800 nm. The corresponding dynamic response characteristics were measured by applying a square-wave voltage alternating between 55 and 100 V (Fig. 6d). The graph shows a decrease in the displacement amplitude as the frequency increases, indicating that the modulator has a critical damping or overdamping response. Moreover, the extinction ratio shows a decreasing trend with increasing frequency, indicating a decline in the optical performance at higher frequencies. Below the specific frequency of 250 kHz, both the displacement and extinction ratio remain relatively constant, indicating that the modulator can effectively operate and achieve nearly full modulation.

**Fig. 7:**
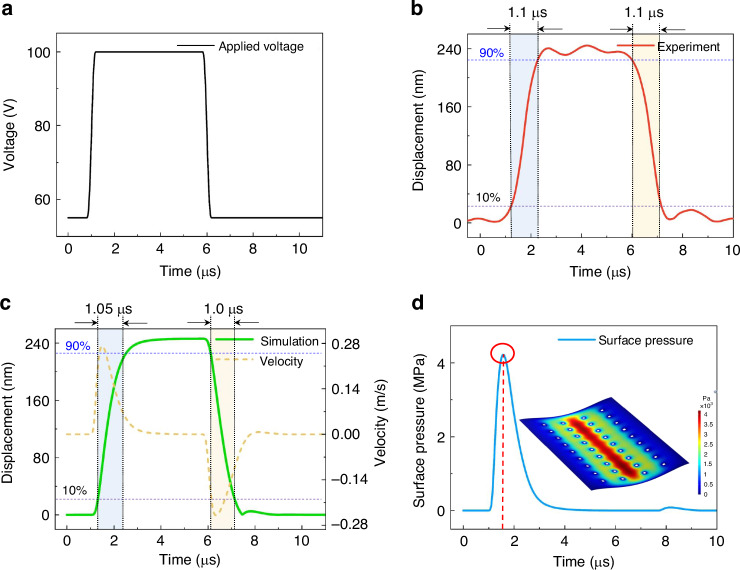
Dynamic temporal response of the MEMS grating modulator. **a** 100 kHz square wave actuation signal (55 to 100 V) for the MEMS grating modulator. **b** The measured mechanical response time agrees well with **c** the simulation results, and the absence of residual mechanical oscillations indicates a critically damped response during state transitions. **d** Simulated pressure distribution on the bottom surface of the grating. The inset shows the instantaneous pressure along the centerline of the surface during transitions

As an example, we conducted a temporal response measurement of the modulator using a square-wave signal with a frequency of 100 kHz. Owing to the load capacity of the voltage amplifier at 100 kHz, the generated waveform is not an ideal square wave, with a slew rate of 0.3 μs (Fig. 7a). The measured temporal mechanical response of the modulator in Fig. 7b shows that a displacement of 240 nm is achieved under the square wave. Both the rise and fall mechanical settling times of the modulator are ~1.1 μs. The mechanical response does not exhibit redundant mechanical oscillations, which implies that the modulator exhibits a critical damping response^43^. This critical damping response not only shortens the transition time but also prevents the mechanical hysteresis of the modulator. To further validate the experimental results and investigate the observed critical damping response, a numerical FEM simulation was conducted. Figure 7c presents the simulation results for the displacement and velocity as functions of time, along with the applied voltage parameters in the simulation, which are consistent with those in the experiment. The simulated mechanical response is in good agreement with the experimental results, with a displacement of 247 nm and a rise time of 1.05 μs. The motion velocity of the moving grating first sharply increases and then monotonically decreases to near zero, which indicates that mechanical settling without redundant oscillation occurs during the rising and falling transitions. To verify whether air damping predominantly influences this dynamic response, Fig. 7d shows the pressure variation on the lower surface during the movement of the grating pitch and the pressure distribution of a single grating pitch. When the grating surface is energized to move downwards, a portion of the air is discharged from the release hole, but the compressed air between the grating surface and substrate can induce an appropriate squeeze-damping force. Owing to its fast movement velocity, each grating surface experiences a maximum instantaneous pressure (or damping force) of about 4 MPa along its centerline.

**Fig. 8:**
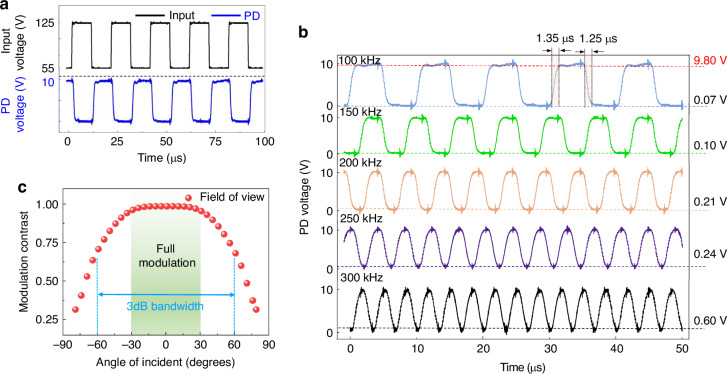
Optical modulation performance of the MEMS grating modulator. **a** Input signal from the amplifier (black) and output signal from the PIN PD at near-normal incidence (blue). **b** Detected voltage signal of the zeroth-order beam at operating frequencies from 100 to 300 kHz. **c** Modulation contrast variation with the incident angle at an operating frequency of 100 kHz

Figure 8a shows the input voltage signal (in black) and the detected signal (in green) of the zeroth-order beam measured by a PIN photodetector (PD) under near-normal incidence at 1550 nm. The input signal consists of a 55 kHz square wave with a 50% duty cycle, and amplitudes of 55/125 V were applied to achieve the maximum modulation contrast at the test wavelength of 1550 nm. The PIN photodetector-detected voltage (PDV) signal shows strong consistency with the input signal, with the maximum and minimum PDVs being detected at 55 and 125 V, corresponding to the “on” and “off” states of the device. The detected peak and base values of the voltage signal for the 0^th^-order beam are 9.8 and 0.07 V, respectively. The modulation contrast was calculated using the Michelson peak-to-peak contrast as follows:

4 $${{\mathrm{MC}}}=\frac{({{{\mathrm{PDV}}}}_{\max }-{{{\mathrm{PDV}}}}_{m{in}})}{({{{\mathrm{PDV}}}}_{\max }+{{{\mathrm{PDV}}}}_{m{in}})}$$

The calculated modulation contrast for the proposed modulator exceeds 98% at normal incidence. Furthermore, the rapid mechanical response time enables the modulator to operate at elevated frequencies. Figure 8b shows the measured PD signal of the zeroth-order beam under frequencies from 100 to 300 kHz. The measured optical rise/fall times at 100 kHz are 1.35 and 1.25 μs. The increased response time compared with the result in Fig. 8b is due to testing of the optical modulation performance at 1550 nm, at which the higher modulation depth requires the device to undergo greater displacement. Notably, the PDV signal presents a square wave within a frequency of 200 kHz. As the frequency further increases, the signal waveform gradually changes to a sine wave, which is attributed to the mechanical response time of the modulator being insufficient to match the rapid rate of signal variations. However, frequencies from 100 to 250 kHz demonstrate exceptional modulation capabilities, with modulation contrasts exceeding 95%. The modulation contrast decreases at 250 kHz due to a slight reduction in the displacement amplitude at higher frequencies, which can be attributed to the amplitude‒frequency characteristics of the modulator. Nevertheless, a modulation contrast of 88.4% can still be achieved at 300 kHz. Figure 8c illustrates the modulation contrast variation with the incident angle, showing that the proposed modulator can achieve nearly full modulation within an incident angle range of ±30° and a field of view (FOV) of 120° with a 3 dB bandwidth (full width at half-maximum) at 100 kHz. To provide a better understanding of the robustness of our device under various operational conditions, we have performed the device of extinction ratio with varying voltage under different working temperatures and incident angles, see Figs. S1, S2 in Supplementary Information.

## Discussion

For optical modulators applied in FSO communication or remote sensing applications, the aperture size and efficiency are crucial factors for improving the detection range and energy efficiency. In this work, we introduced a tunable sinusoidal grating-based MEMS grating modulator with a large-scale extendable aperture and high optical efficiency. The proposed modulator is constructed with a tunable grating pitch array, in which each pitch is designed with a broadside-constrained continuous ribbon to generate deflection deformation. Owing to the unidirectional equal stiffness of the pitch, its resonant frequency remains constant regardless of variations in its longitudinal dimensions. Consequently, a large-scale aperture can be constructed by extending the pitch length and arranging the pitches in a one-dimensional array. Additionally, a continuous sinusoidal grating profile, rather than a rectangular discrete grating, was adopted to modulate the diffraction intensity with a higher optical efficiency. Owing to the proposed sinusoidal grating structure, the modulator chip can be efficiently fabricated with only a two-layer structure configuration. For the current embodiment, the implemented continuous sinusoidal grating enables the modulator to have a surface fill factor of over 96% and an optical efficiency of over 90%, corresponding to an optical insertion loss of less than 1 dB. The end-free grating allows scalability along the grating, enabling a large aperture of 30 × 30 mm. Regarding optical modulation performance, an extinction ratio of over 20 dB and a modulation contrast of over 98% are achieved for the 0^th^-order beam with normal incidence light. The reported modulator can achieve a dynamic modulation contrast of over 95% within a 250 kHz operating frequency. The proposed modulator also achieves nearly full modulation within an incident angle range of ±30° and an FOV of 120° under a 3 dB bandwidth.

This study introduces a novel strategy to expand the aperture and increase the efficiency of MEMS optical modulators with a two-layer configuration. To fabricate a MEMS grating modulator with a two-layer configuration, one of the most obvious ways is to utilize an SOI wafer. Here, we employed a double mask etching process followed by a wet etching process to fabricate the device. The movable ribbons within each pitch exhibit upward buckling deformation after the oxide layer is released, which may be attributed to the residual compressive stress in the device layer of the SOI wafer from the fabrication process. In terms of long-term performance, the intrinsic stress in the SOI device layer has minimal impact on the lifetime and stability of our device. According to refs. ^44,45^, the breaking stress of single-crystal silicon is approximately above 1.0 GPa^44,45^, and the fatigue stress in single-crystal silicon-based MEMS devices is about 3.0 GPa^46,47^. However, reported works show that the intrinsic stress in the SOI device layer is typically less than 40 MPa^48,49^, so this magnitude of intrinsic stress can be neglected when assessing the long-term lifespan of the device. The upward buckling deformation due to intrinsic stress in the SOI device layer makes the proposed modulator to be an unwanted surface. To address this, an applied initial electrostatic force recovers this unwanted surface into a desired flat surface to achieve “on” state modulation. However, the buckling deformation may still have a minor impact on the optical modulation performance of our device. The initial upward buckling deformation could cause the movable ribbons to elongate. Under actuation, they may not retain a perfect planar configuration but instead exhibit a quadratic surface after returning to the “on” state, which could decrease the zeroth-order diffraction efficiency. Since the stress in SOI originates from its fabrication process, this intrinsic stress cannot be effectively eliminated. However, we have implemented some methods to alleviate or prevent further impacts of stress on our device. For example, an appropriate thickness of gold film was deposited on the SOI device layer to help balance the residual compressive stress. This helped prevent the buckling deformation caused by compressive stress from increasing significantly.

After measurements with various temperatures, no significant impact on the optical performance of the device was observed due to possible thermal expansion or variations in structural materials from high-temperature environments (Fig. S1 in the Supplementary Information). However, a high-humidity environment can cause stiction failure during long-term operation. In such conditions, water vapor would accumulate in the gaps between the grating surface and the substrate. The long-term exposure to high humidity could potentially affect the performance and cause device failure. Moreover, high humidity may affect the electrical stability of the device. Humidity-induced migration of water molecules can create conductive pathways and degrade the insulating properties of the air gap, causing leakage currents and increasing the risk of electrical breakdown. To address this issue, we can employ hermetic sealing in conjunction with desiccants to maintain a dry operating environment for the movable gratings. In addition to the hermetic sealing approach, we can also deposit a layer of Parylene coating over the entire device to achieve electrical insulation and humid protection^50^.

In our device, the only moving mechanical element is the ribbon which is made from single-crystal silicon of the SOI device layer. Under actuation, the stress within ribbons is less than 5% of the breaking stress of single-crystal silicon. Specifically, the breaking stress of single-crystal silicon is almost above 1 GPa^44,45^ while the maximum stress in our device during operation is less than 50 MPa. Moreover, the fatigue stress in single-crystal silicon-based MEMS devices is about 3 GPa^46,47^ according to reported works. However, our device has a maximum stress of less than 50 MPa and an electrostatic force load within 1 μN, which is much smaller than the reported fatigue stress. Therefore, this magnitude of stress can be neglected when assessing the long-term lifespan of the device and it can be considered as having an infinite cycle life. Furthermore, the ribbons operate in a non-contact mode with relatively small displacement. To illustrate, if a grating ribbon is scaled up so that its length is 1.0 m, the displacement for full modulation will be less than 4 mm. Additionally, since the ribbons never experience contact, they are not subject to wear issues. These intrinsic reliabilities enable our device to have good repeatability and lifetime. Long-term lifetime testing of our device will be conducted in subsequent work.

Taken together, the modulator featuring a tunable sinusoidal grating presented in our work provides a proof of concept and an effective strategy for enhancing the aperture size and optical efficiency of MEMS-based optical modulators. The current modulator design has the potential to further enhance the usability of modulating retroreflector (MRR)-FSO systems with larger FOVs and high-speed optical attenuation applications. Based on this tunable sinusoidal grating modulation concept, expanded features and various devices can potentially be realized and developed in the future. For example, by employing electrical insulation channel isolation, multichannel control can be achieved, enabling independent control of the reflected light intensity of each individual grating pitch. Moreover, the design of a grating arrangement with periodic odd and even variations can offer versatile reflected beam-shaping capabilities and further expand the functionality of the modulator.

## Materials and methods

### Fabrication and assembly of the device

The fabrication of the MEMS grating modulator began with a commercial SOI wafer (Suzhou Rdmicro Co., Ltd), consisting of a 1-μm device layer, a 3-μm buried oxide layer, and a 400-μm silicon substrate. A 5 nm adhesive Cr film and an Au film with thicknesses of less than 50 nm were deposited in sequence on the SOI device layer via physical vapor deposition (Kurt J. Lesker PVD 75). A contact lithography system (SUSS MA6) was used to pattern a resist layer (S1813) spun on top of the device layer. A portion of the Au film was wet-etched with ultrasonication to expose the silicon pattern area for subsequent dry etching. Subsequently, dry etching was performed via ICP etching (SPTS Rapier) to form holes deep in the buried oxide layer. Following this step, the whole wafer was laser-diced into several chips for a single wet etching release. Finally, the buried oxide layer of the standalone chip was partially etched with 49% hydrofluoric acid. Following fabrication, the modulator chips were mounted and wire bonded to custom PCBs via conductive silver adhesive bonding (IC-01) and gold ball bonding.

### Measurement of the electromechanical and optical performance

An LDV featuring a 532 nm laser with a minimum focused beam of 3 μm was used to measure the electromechanical performance of the modulator. The input signals were generated by an arbitrary waveform generator (Keysight 33500B, 1 GSa/s) and amplified by a high-voltage amplifier (Pintech HA-405), which featured a bandwidth of up to 1 MHz and a maximum output voltage of 200 V. The optical modulation performance was evaluated under the optical test setup illustrated in Fig. 7b in ambient air at room temperature. At the transmitter, laser modules (Jcoptix, MD10 series) were used as the 635 nm wavelength and 1550 nm wavelength laser sources. Two collimating lenses with focal lengths of 30 and 150 mm were mounted on the conjugate focal plane to expand the point light into a plane wave. Following the collimating lenses, the first 50/50 beam splitter was mounted at a 45-degree angle to transmit the input plane wave and reflect the diffraction beams from the modulator. The second 50/50 beam splitter was placed parallel to the first beam splitter to project the far-field pattern onto a board and reflect the modulated beam into the detector. To prevent the high-order diffraction spots from overlapping, the distance from the projection board and detector to the first beam splitter was at least 150 cm. An aperture diaphragm was mounted in front of the detector to block all the high-order beams except for the zeroth-order beam. The light signals were subsequently detected by a 1 MHz PIN photodiode (Circle Electronics, TO-46), and an I-V transfer circuit (Techlife, IV-AMP1656SP) was employed to convert the photocurrent into a voltage signal. Following this step, the voltage signal was digitized by a real-time oscilloscope (Keysight DSOX3024T, 5 GSa/s sampling rate, 200 MHz analog bandwidth). An optical power meter (Daheng Optics, GCI-080201) was used to measure the power of the 0th-order beam under quasistatic conditions.

## Supplementary information

Supporting file
