# Supplementary material for: A MEMS grating modulator with a tunable sinusoidal grating for large-scale extendable apertures
Source: Microsyst Nanoeng. 2025 Mar 3;11:39. doi: 10.1038/s41378-025-00894-7 (PMC11873166; doi:10.1038/s41378-025-00894-7)
Supplement: Supplementary file 1 — Supporting file [file 41378_2025_894_MOESM1_ESM.docx]

**Supplementary information**

A MEMS grating modulator with a tunable sinusoidal grating for large-scale extendable apertures

Datai Hui^,1^ Dongpeng Li^,1^ Binbin Wang^,1^ Yongqian Li^1,2, *^ Jiaqian Ding^, 1^ Laixian Zhang^3^ and DAYONG QIAO ^1,2^

^1^Key Laboratory of Micro/Nano Systems for Aerospace, Ministry of Education, Northwestern Polytechnical University, Xi'an 710072, China

^2^Research & Development Institute of Northwestern Polytechnical University, Ningbo，315103, China

3Key Laboratory of Intelligent Space TTC&O, Ministry of Education, Space Engineering University, Beijing, 101416

[huidatai@mail.nwpu.edu.cn](mailto:huidatai@mail.nwpu.edu.cn), dongpengli@mail.nwpu.edu.cn, wangbinbin@nwpu.edu.cn, liyq@nwpu.edu.cn, jiaqianding@mail.nwpu.edu.cn, zhanglaixian@126.com，dyqiao@nwpu.edu.cn

Contact details of the corresponding author: [liyq@nwpu.edu.cn](mailto:liyq@nwpu.edu.cn), 086-029-88460353-610

**Table S1**. Comparison of key properties between the sinusoidal grating-based modulator reported here with other relevant works

| MEMS elements type | Optical efficiency | | Device aperture  (mm^2^) | Response time (μs) | Reference |
| --- | --- | --- | --- | --- | --- |
| 1D-Rectangle grating | **＞**80% | 41.0 × 0.15 | | ＜0.2 | [1] |
| 1D-Rectangle grating | **＞**75% | - | | 0.8 | [2] |
| 2D-Rectangle grating | **＞**85% | 3.1 × 3.2 | | 5.7 | [3] |
| 1D-Micromirror array | **＞**87% | - | | 2.0 | [4] |
| 1D-Micromirror array | **＞**90% | 5.0 × 6.0 | | - | [5] |
| Annular Micromirror array | - | ɸ 8.2 | | 64.8 | [6] |
| 2D-Micromirror array | **＞**95% | 26.0 × 26.0 | | 75.0 | [7] |
| 2D-Micromirror array | **＞**90% | 34.5 × 21.6 | | ＜2.0 | [8] |
| **1D-sinusoidal grating** | **＞90%** | **30.0 × 30.0** | | **＜1.5** | **This work** |

Table S1 lists the key properties of the sinusoidal grating-based modulator reported here and other relevant works. These properties include the optical efficiency, device aperture and response time, which are generally used to characteristic MEMS modulators. The rectangle grating based modulator can achieve a fast response time of less than 1.0 μs^1,2^, but the device apertures are limited to tens of square millimeters^1,2,3^, and the optical efficiency can hardly reach 80% due to their discrete grating surface^1,2^. Typical micromirror array-based modulators can offer an optical efficiency over 90%^5-8^ and achieve a response time within 2.0 μs^4,8^. A large-scale aperture over hundred square millimeters can be realized by 2D arranging the mirror unit^7,8^. However, the scalability faces challenges due to its sophisticated fabrication process. Benefiting from the proposed tunable sinusoidal grating with continuous surface, our proposed modulator has a surface fill factor of over 96% and an optical efficiency of over 90%. Instead of end-constrained movable ribbons, we constrain the MEMS grating modulator through broadside-constrained continuous ribbons. This allows scalability along the grating, enabling the device aperture is significantly expanded to a few hundred square millimeters. Meanwhile, due to the ribbon's unidirectional equal stiffness, its resonant frequency remains constant regardless of variations in longitudinal dimensions, enabling the device to achieve a fast response time within 1.5 μs.


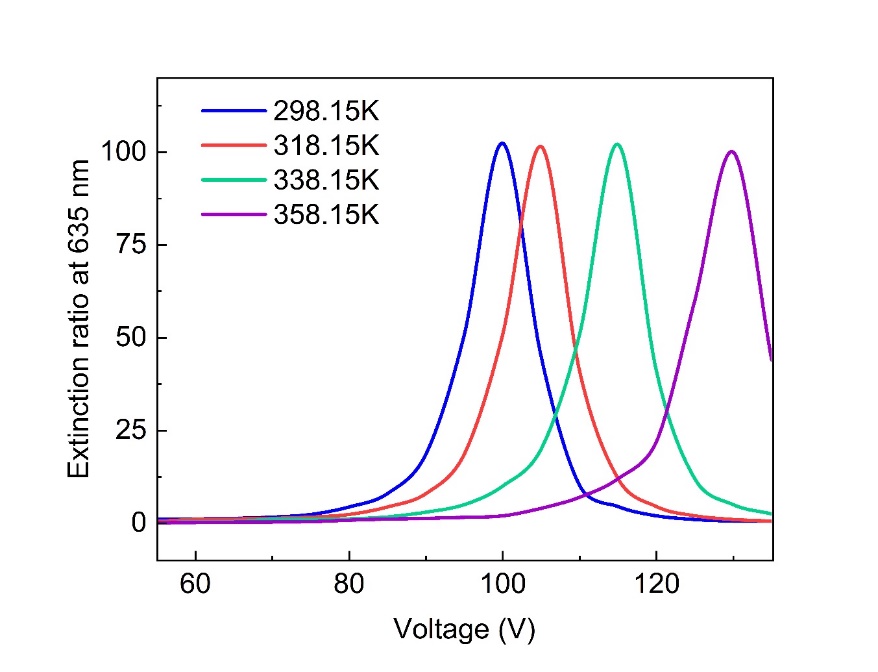


**Fig.S1**. Extinction ratio (tested at a wavelength of 635 nm) with varying applied voltage under different working temperatures.

Fig. S1 presents the extinction ratio at 635 nm as a function of applied voltage for four representative operating temperatures (298.15 K, 318.15 K, 338.15 K, and 358.15 K). Each curve exhibits a well-defined peak, demonstrating that the device can achieve a nearly identical extinction ratio of around 100 across all tested temperatures by suitably adjusting the voltage. Notably, as the temperature increases, achieving the same level of extinction ratio requires incrementally voltage shift. This increase may be attributed to enhanced buckling deformation of the ribbon at elevated temperatures, which enlarges the initial gap and consequently requires a greater applied voltage to attain the same modulation depth.


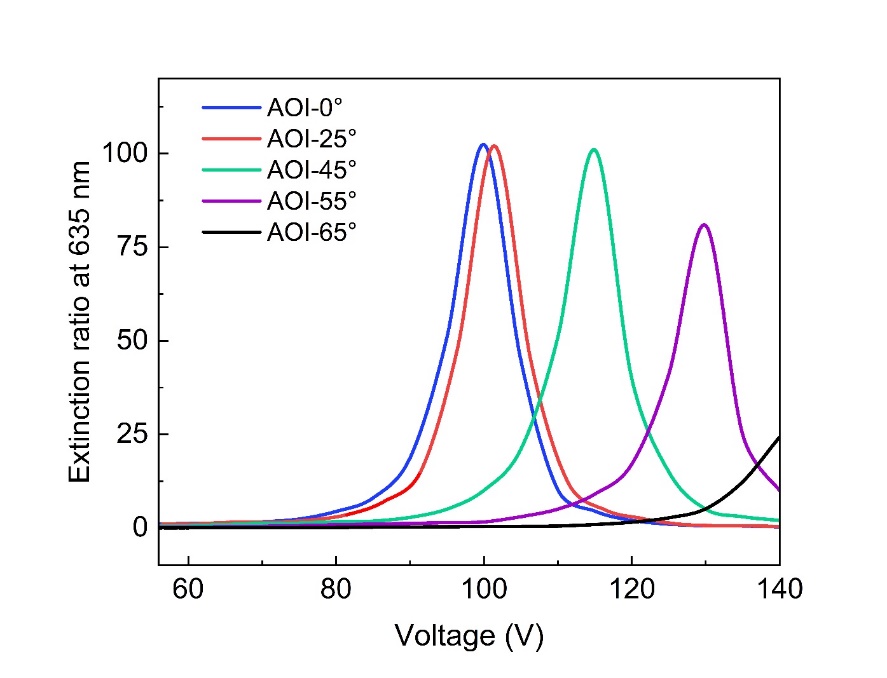


**Fig.S2**. Extinction ratio (tested at a wavelength of 635 nm) with varying applied voltage under different incident angles.

Fig.S2 illustrates the extinction ratio at 635 nm under different angles of incident (AOI) with varying voltages. It is obvious that the extinction ratio of the device is less sensitive to lower incident angles. For example, at incident angles of 0° and 25°, the required voltage shifts to reach the optimal extinction ratio is less than 2 V. As the angle increases to 45°, the voltage to achieve the optimal extinction ratio increases substantially but can still yield extinction ratio reach 100 with an appropriate voltage shift. With further increases in the incident angle, the voltage shift becomes more pronounced, and the maximum attainable extinction ratio has a slight decline. For instance, at incident angle of 55°, the extinction ratio is approximately 25% lower than at normal incidence. At an incident angle of 65°, the device does not reach its maximum extinction ratio within the applied voltage for preventing the pull-in effect caused by further increased voltage.

**Reference**

1. Amm, D. T. & Corrigan, R. W. Optical performance of the grating light valve technology. *Projection Displays V*. **3634**, 71-78 (1999).
2. Yun, S. K., Song, J., An, S., Yeo, I. & Choi, Y. E. A novel diffractive micro-optical modulator for mobile display applications. *MOEMS and Miniaturized Systems VII* .**6887**, 17-27 (2008).
3. Wang, Y., Zhou, G., Zhang, X., Kwon, K. & Blanche, P. A. 2D broadband beamsteering with large-scale MEMS optical phased array. *Optica*. **6**, 5 (557-562).
4. Wang, Y., Wu, M. C. Micromirror based optical phased array for wide-angle beamsteering. *IEEE 30th International Conference on Micro Electro Mechanical Systems*. 897-900, 2017.
5. Xiao, X., Mao, T., Shi, Y., Zhou, K. & Hao, J. A new fabrication method for enhancing the yield of linear micromirror arrays assisted by temporary anchors. *Microsyst Nanoeng*. **10**, 63 (2024).
6. Ersumo, N. T., Yalcin, C., Antipa, N., Pégard, N. & Muller, R. A micromirror array with annular partitioning for high-speed random-access axial focusing. *Light: Science & Applications*. **9**, 1, 183 (2021).
7. Bifano T. MEMS deformable mirrors. *Nature Photonics*. **5**, 21-23 (2011).
8. Texas Instruments. *DLP 0.8 WUXGA series 800 DMD* (2024).
